# Complex host-pathogen coevolution in the *Apterostigma *fungus-growing ant-microbe symbiosis

**DOI:** 10.1186/1471-2148-6-88

**Published:** 2006-11-03

**Authors:** Nicole M Gerardo, Ulrich G Mueller, Cameron R Currie

**Affiliations:** 1 Section of Integrative Biology, University of Texas at Austin, Austin, TX, USA; 2 Smithsonian Tropical Research Institute, Apartado 2072, Balboa, Republic of Panama; 3 Department of Ecology and Evolutionary Biology, P.O. Box 210088, University of Arizona, Tucson, AZ, USA; 4 Department of Bacteriology, University of Wisconsin at Madison, Madison, WI, USA

## Abstract

**Background:**

The fungus-growing ant-microbe symbiosis consists of coevolving microbial mutualists and pathogens. The diverse fungal lineages that these ants cultivate are attacked by parasitic microfungi of the genus *Escovopsis*. Previous molecular analyses have demonstrated strong phylogenetic congruence between the ants, the ants-cultivated fungi and the garden pathogen *Escovopsis *at ancient phylogenetic levels, suggesting coevolution of these symbionts. However, few studies have explored cophylogenetic patterns between these symbionts at the recent phylogenetic levels necessary to address whether these parasites are occasionally switching to novel hosts or whether they are diversifying with their hosts as a consequence of long-term host fidelity.

**Results:**

Here, a more extensive phylogenetic analysis of *Escovopsis *lineages infecting the gardens of *Apterostigma *ants demonstrates that these pathogens display patterns of phylogenetic congruence with their fungal hosts. Particular clades of *Escovopsis *track particular clades of cultivated fungi, and closely-related *Escovopsis *generally infect closely-related hosts. Discordance between host and parasite phylogenies, however, provides the first evidence for occasional host-switches or acquisitions of novel infections from the environment.

**Conclusion:**

The fungus-growing ant-microbe association has a complex coevolutionary history. Though there is clear evidence of host-specificity on the part of diverse *Escovopsis *lineages, these pathogens have switched occasionally to novel host fungi. Such switching is likely to have profound effects on how these host and parasites adapt to one another over evolutionary time scales and may impact how disease spreads over ecological time scales.

## Background

Most parasites are intimately dependent on one or a few hosts. Because of this host fidelity, parasites are expected to track speciating hosts by speciating themselves. This process, known as cospeciation, will lead to cocladogenesis, the topological matching of symbiont phylogenies. Parasite and host phylogenies are rarely identical, however; forces such as duplication (parasite speciation in the absence of host speciation), sorting events (host speciation without commensurate parasite speciation), and host-switching (parasites begin to use a new host) [1,2] can generate discordance between the phylogenies of hosts and their symbionts. Despite these complications, congruent phylogenies are known in host-parasite associations [3-5] and in host-mutualist associations as well [6-8].

The fungus-growing ant-microbe symbiosis is a novel example of a system in which cocladogenesis occurs between a host and both its mutualistic and parasitic symbionts. Research over the last decade has demonstrated the congruence of the phylogenies of fungus-growing ants, the fungi that they cultivate (i.e., their fungal cultivars) and the cultivar-attacking pathogen *Escovopsis *at ancient phylogenetic levels [9-11]. Genetic analyses of more recently diverged, younger lineages demonstrate discrepancies between ant and cultivar associations, which are likely due to a combination of lateral transfer of cultivars between colonies and occasional domestication of free-living fungi by the ants [12-15]. To date, the two published phylogenetic studies of the *Escovopsis*-cultivar association indicate no discordance between the phylogenies of the cultivars and *Escovopsis *[10,16].

**Figure 1:**
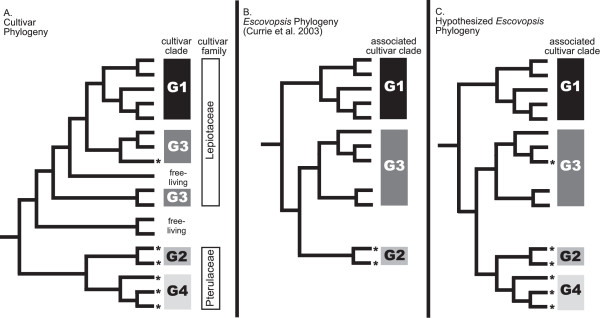
**Current symbiont phylogenies and hypothesized *Escovopsis *relationships**. (a) Cultivar phylogeny simplified from [9-11] (b) *Escovopsis *phylogeny from [10]. This phylogenetic reconstruction, the most complete to date, includes very few *Apterostigma*-associated pathogens. (c) Hypothesized *Escovopsis *phylogeny in which there are four distinct *Escovopsis *clades corresponding to the four known cultivar clades. In this hypothesized phylogeny, the Pterulaceae-attacking pathogens are distinct from the Lepiotiaceae-attacking pathogens. * indicates *Apterostigma*-associated symbionts. Note that the only *Apterostigma*-associated symbionts outside the G2/G4 clade are those isolated from colonies of *A. auriculatum*, the only *Apterostigma *sp. that does not cultivate pterulaceous fungi [11, 13]. See introduction for further details.

Ancient codiversification of fungus-growing ants and their cultivars is driven by the intimate dependence of the ants on fungus as their primary food source and the intimate dependence of the fungus on ants for protection, nutrition and dispersal [17]. In ants, the ability to cultivate fungi for food arose only once, about 50–60 million years ago, and gave rise to roughly 200 described, extant species of fungus-growing ants (Tribe Attini)[18]. The long coevolutionary history of these mutualists has led to the specialization of each ant species on the cultivation of a unique, narrow range of cultivated fungi, most of which are in the family Lepiotaceae. As depicted in Figure 1A, these lepiotaceous cultivars form two morphologically and molecularly distinct groups ('G1' and 'G3'; [9]). There has been one switch to a distantly related cultivar; most ants in the genus *Apterostigma *now cultivate fungi in the family Pterulaceae [19], which is distantly related to the family Lepiotaceae. The pterulaceous cultivars fall into two monophyletic, morphologically distinct cultivar groups ('G2' and 'G4' in Figure 1A; [11]). One *Apterostigma *species, *A. auriculatum*, has retained the ancestral state of growing lepiotaceaous cultivars [11].

Currie et al. [10] demonstrated that, at ancient levels, the phylogeny of *Escovopsis *(Ascomycota: Hypocreales) (Figure 1B), a genus of specialized, highly pathogenic microfungi that attack the ants' fungal cultivars, matches that of the ants' diverse cultivars and consequently that of the ants themselves. *Escovopsis *has only been found associated with nests of attine ants. Upon establishing infection, *Escovopsis *consumes the ants' cultivated fungi and can devastate attine colonies [20-22]. Though infection rates vary across host species, infections are prevalent in colonies of many attine genera throughout their geographic ranges [16,20,21]. *Escovopsis *is thought to track the cultivars because of the coevolutionary specialization of each *Escovopsis *lineage on attacking and overcoming defenses of only a narrow range of cultivar hosts [16,23].

Ancient phylogenetic congruence between cultivars and *Escovopsis *suggests that these pathogens may be tightly tracking their speciating hosts by speciating themselves, and that *Escovopsis *lineages have not switched to novel cultivar hosts over evolutionary time. To test for host-switching, however, it is necessary to include extensive sampling across the diversity of both hosts and symbionts. Previous studies of *Escovopsis *host-fidelity have included few samples of *Apterostigma*-associated *Escovopsis *despite the fact that they are an extremely diverse group of fungus-growing ant pathogens. Currie et al. [10], the most extensive phylogenetic analysis of *Escovopsis *to date, included only two *Apterostigma*-associated *Escovopsis*, which were morphologically similar and were isolated from ant colonies that raised closely-related fungi. Not surprisingly, these isolates formed a single monophyletic "*Apterostigma Escovopsis*" clade (Figure 1B). However, unlike the other fungus-growing ant genera, which each raise cultivars in a single cultivar group, *Apterostigma *ants raise cultivars in three groups (G2, G3 and G4 in Figure 1A), which are each attacked by morphologically distinct *Escovopsis *types[23]. More extensive sampling of these diverse *Apterostigma *pathogens, therefore, can reveal the extent to which *Escovopsis *species are host-faithful, tracking their particular hosts without host-switching.

Through extensive geographic sampling and phylogenetic analysis of *Apterostigma*-associated *Escovopsis*, we ask whether host and pathogen phylogenies are still congruent when genetic analyses are extended to include the diversity of the *Apterostigma*-associated *Escovopsis*. First, do *Apterostigma*-associated *Escovopsis *form a monophyletic clade as the *Apterostigma *ants do, or are *Apterostigma*-associated *Escovopsis *polyphyletic like their cultivars? Second, do the *Apterostigma Escovopsis *form three distinct clades that correspond to the three cultivar groups (G2, G3 and G4) raised by the different species of *Apterostigma *ants? Based on earlier findings that *Escovopsis *is highly cultivar-type specific [16], we hypothesize that more extensive sampling will reveal that the *Apterostigma*-associated *Escovopsis *are not monophyletic like their associated ant-hosts, because the *Escovopsis *that infects lepiotaceous *Apterostigma *cultivars (i.e. the cultivars raised by *A. auriculatum*) will be more closely-related to *Escovopsis *isolated from lepiotaceous gardens of non-*Apterostigma *ant species than to *Escovopsis *isolated from pterulaceous *Apterostigma *gardens (Figure 1C). This would support findings of Currie et al. [10], depicted in Figure 1B, that the pterulaceous-attacking *Escovopsis *form a monophyletic clade distinct from the *Escovopsis *that infects lepiotaceous cultivars, but would contradict their findings of complete congruence between ant, cultivar, and *Escovopsis *phylogenies. We further hypothesize that more extensive sampling will reveal that pterulaceous-attacking *Escovopsis *will fall into two clades associated with the two pterulaceous cultivar groups (G2 and G4). Overall, in looking at the fungus-growing ant-microbe symbiosis as a whole, we predict four host-specific *Escovopsis *clades that are each specialized at attacking one of the four known fungus-growing ant cultivar groups (G1, G2, G3 and G4) (Figure 1C).

## Results

### Diversity of *Apterostigma*-associated *Escovopsis*

Of 623 colonies from which microbes were sampled, at least one fungal symbiont (either cultivar or *Escovopsis*) was isolated from each of 410 colonies. For the purpose of this study, based on field identification of the ants, garden architecture and growth form of cultivar isolates, each colony was classified as either a G2, G3 or G4 colony, which raise respectively G2, G3 and G4 cultivars (see introduction).

*Escovopsis *infection of these 410 colonies was common and pathogen phenotypes were diverse. G2 colonies and G4 colonies had much higher infection rates than G3 colonies (G-test with Yate's Correction: G2 vs. G3, G = 36.0, df = 1, p < 0.0001; G4 vs. G3, G = 6.3, df = 1, p < 0.0001; G2 vs G4, G = 0.1, df = 1, p = 0.8). More than 50% of G2 and G4 colonies were infected with at least one *Escovopsis *type, whereas only 11% of G3 colonies were infected (Table 1). *Escovopsis *samples isolated from infected colonies were classified into four morphotypes based on spore-color: brown, yellow, white and pink. These types have different micromorphological conidiophore structures (Currie, unpublished) and likely represent different *Escovopsis *lineages. In the absence of proper species descriptions, we will refer to the different *Escovopsis *lineages by their characteristic spore-color (white, pink, yellow, brown). While white and pink *Escovopsis *were each specific to a single cultivar group, brown *Escovopsis *infected both G2 cultivars and G4 cultivars, and yellow *Escovopsis *infected both G2 cultivars and G3 cultivars. The yellow *Escovopsis *isolates associated with these two clades, however, are micromorphologically distinct from one another (Currie, unpublished) and likely are two separate species. A small percentage of colonies (10% of G2 colonies) were infected by multiple *Escovopsis *morphotypes (Table 1).

**Table 1:** Distribution and diversity of *Apterostigma Escovopsis *infections.

Cultivar Clade	# colonies successfully sampled for symbiotic microbes	# colonies infected with *Escovopsis*	# colonies infected with...				# colonies infected with multiple *Escovopsis *types
				
			brown *Escovopsis*	yellow *Escovopsis*	white *Escovopsis*	pink *Escovopsis*	
G2	350	185 (52%)	141	64^2^	17	0	35^1^
G3	55	6 (11%)	0	1^2^	0	5	0
G4	6	4 (67%)	4	0	0	0	0

*Escovopsis *types are identified here according to spore-color. ^1 ^For the G2-cultivar colonies, the number of infections based on spore-color exceeds the number of total infections because many colonies were infected with two or three *Escovopsis *types. ^2 ^Though sharing a similar spore-color, the G2-attacking and G3-attacking yellow *Escovopsis *have distinct micromorphologies (Currie, unpublished).

### Phylogenetic relationships of *Apterostigma*-associated *Escovopsis*

**Figure 2:**
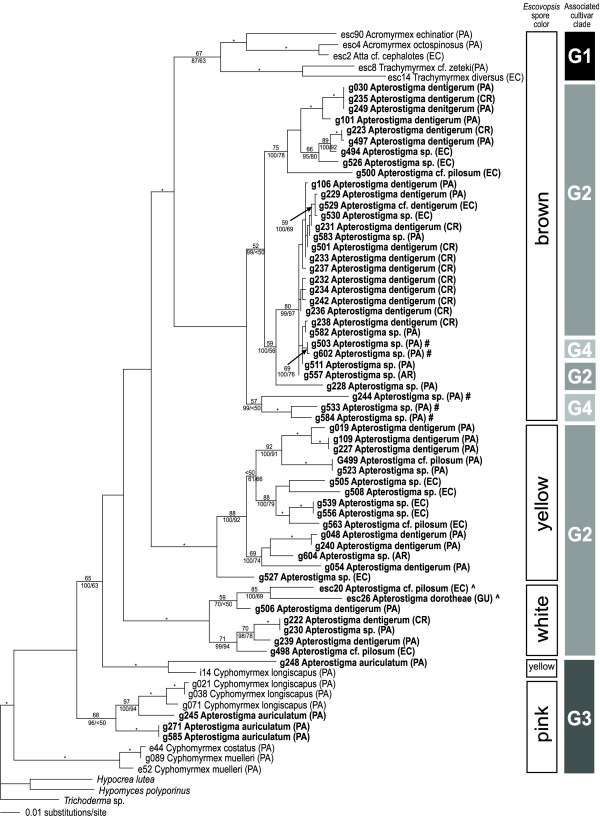
***Escovopsis *phylogeny based on EF-1 alpha sequencedata**. Each branch is labeled with likelihood bootstrap values (above), Bayesian posterior probabilities (below, left) and parsimony bootstrap values (below, right). Unlabeled branches have values of less than 50 for at least two analyses of support. * indicates that all three support values are 95 or greater. Each *Escovopsis *node is labeled with a sample code, the name of the associated ant species, and the country of origin (AR, Argentina; CR, Costa Rica; GU; Guyana; EC, Ecuador; PA, Panama). Labels for isolates from *Apterostigma *colonies are in bold. # emphasizes the G4-associated *Escovopsis*, which are not monophyletic. ^ marks the two *Apterostigma Escovopsis *isolates included in a previously published analysis [10]. Bars along the right side indicate the spore-color of the sample and the host-cultivar clade. Not all outgroups are shown for clarity.

The results of parsimony, likelihood and Bayesian analyses were highly concordant. Three well supported clades were identified that correspond to brown, white and pink *Escovopsis *(Figure 2). Yellow *Escovopsis *is not monophyletic; G2-attacking yellow *Escovopsis *is genetically distinct from the single isolate of G3-attacking yellow Escovopsis. Overall, as predicted, these diverse *Apterostigma*-associated *Escovopsis *do not form a monophyletic clade. Both the yellow and pink *Escovopsis *isolated from *Apterostigma *colonies with G3 cultivars are nested within other G3-attacking *Escovopsis *and are distinct from other *Apterostigma*-associated *Escovopsis*. Contradictory to our predictions, G2-attacking and G4-attacking *Escovopsis *do not form separate, monophyletic clades. Within the brown *Escovopsis*, there are two clades of G4-associated *Escovopsis*. Parametric-bootstrapping verified the polyphyly of isolates of G4-associated *Escovopsis*. The null hypothesis of a single origin of G4-associated *Escovopsis *was rejected at p < 0.001. This implies that brown *Escovopsis *has switched multiple times between G2 and G4 hosts.

## Discussion

**Figure 3:**
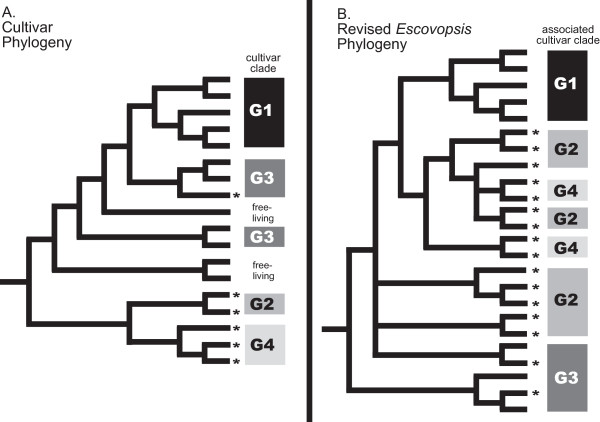
**Comparison of cultivar and *Escovopsis *phylogenies**. (a) Cultivar phylogeny as in Figure 1A. (b) *Escovopsis *phylogeny synthesized from Figure 2. * indicates fungal symbionts from *Apterostigma *colonies. Clades of *Escovopsis *corresponding to cultivar clades suggests coevolutionary specialization of the pathogen, but discordance of the host and pathogen phylogenies as a whole suggests occasional host-switching by *Escovopsis *during the evolutionary history of the association.

Phylogenetic patterns of the fungus-growing ant microbe symbiosis reveal a coevolutionary history of host-fidelity punctuated by occasional host-shifts. All known *Escovopsis *lineages have some limitation to their host-range. For example, we here show that pink *Escovopsis *attacks only lepiotaceous G3 cultivars (including *A. auriculatum*'s cultivars), white *Escovopsis *attacks only G2 cultivars, and though *Escovopsis *with yellow spores attacks both G2 and G3 cultivars, the yellow *Escovopsis *lineages associated with each of these host groups are morphologically and genetically distinct (Figure 2). Despite this specificity, however, there is not complete congruence of host and pathogen phylogenies as suggested by previous studies [10], indicating that *Escovopsis *host ranges have shifted and may continue to shift (Figure 3). This complex history parallels that of other symbiotic associations in which extensive sampling reveals that codiversification is interrupted often by host-switches [13,24-26]. In fact, it appears that the cases where cocladogenesis persists over evolutionary time are mostly vertically-transmitted endosymbionts [27-30], whereas most ectosymbionts, such as *Escovopsis *and the fungal cultivar, show patterns of switching and absence of strict cocladogenesis with their hosts.

Adaptive processes may explain the host-fidelity of most *Escovopsis *types, which leads to the host-specific *Escovopsis *clades revealed here. Gerardo et al. [23] demonstrated through microbial bioassays that *Escovopsis *lineages are attracted to chemical signals released by their host cultivars. For example, in microbial bioassays, isolates of yellow *Escovopsis *from G2-*Apterostigma *colonies grow more rapidly towards chemical signals produced by G2 than by G4 and G3 cultivars, which is consistent with the host-range of yellow *Escovopsis*. Unlike yellow *Escovopsis*, brown *Escovopsis *from G2-*Apterostigma *colonies is equally attracted to G2 and G4 cultivars. It is possible that this host-attraction would make it easier for brown *Escovopsis *to switch between G2 and G4 hosts than it would be for yellow *Escovopsis *to switch between hosts, because brown *Escovopsis *would be equally likely to move through G2 and G4 fungal gardens, find healthy cultivar and establish infection. This is consistent with the phylogenetic results here, where is seems that brown *Escovopsis *has switched between G2 and G4 hosts.

Microbial bioassays have also revealed that cultivars can defend themselves against some *Escovopsis *but not others [23]. G3-*Apterostigma *cultivars can inhibit isolates of both brown *Escovopsis *and G2-associated yellow *Escovopsis *but cannot inhibit isolates of pink *Escovopsis*, possibly explaining why brown and G2-associated yellow *Escovopsis *do not attack G3 cultivars in nature, while pink *Escovopsis *does. Brown and yellow *Escovopsis *are not, however, inhibited by most isolates of G2 and G4 cultivars, explaining why natural infection is possible in these host-parasite combinations. Overall, both cultivar defense against *Escovopsis *and *Escovopsis' *attraction to host cultivars may maintain *Escovopsis' *specialization and prevent rampant host-switching.

Because *Escovopsis *species are host-specific, we hypothesized that wider sampling of *Escovopsis *would reveal four *Escovopsis *clades that correspond to four cultivar and ant clades (Figure 1). However, contradictory to our hypothesis (Figure 1c), G4-associated *Escovopsis *are not monophyletic (Figure 3b). Furthermore, there is a lack of congruence of cultivar and *Escovopsis *phylogenies at deeper nodes (Figure 3). Whereas previous analyses [9-11] have indicated that *Apterostigma *ants, their pterulaceous cultivars and their associated *Escovopsis *are distantly-related to the highly-derived leafcutter ants and their associated microbes (including G1 cultivars), some *Apterostigma*-associated *Escovopsis *lineages, namely brown *Escovopsis*, are sister to the *Escovopsis *attacking G1 cultivars (fig 3b). This suggests that an *Escovopsis *lineage switched between these two distantly-related, ecologically-distinct fungal hosts (i.e. *Apterostigma *colonies and leafcutter colonies).

Discordance of host and pathogen phylogenies suggests that *Escovopsis *lineages have switched hosts over the evolutionary history of their host association, but the available evidence does not allow inference regarding the frequency at which switching occurs. It is also unclear whether switching involves the acquisition of novel *Escovopsis *strains by the ants from their environment, or whether it involves the direct transmission of *Escovopsis *between colonies by some unknown mechanism. Further research on the exact mechanism of *Escovopsis *transmission would be helpful in revealing the likelihood of pathogen exchange between colonies containing distantly-related cultivars.

## Conclusion

Phylogenetic analyses coupled with extensive sampling of host and parasites reveal a more complete picture of the complexity of the *Escovopsis*-cultivar association in colonies of fungus-growing ants, which consists of specialized pathogen species that occasionally switch between distantly-related hosts. Clades of closely-related *Escovopsis *attack specific cultivar groups, causing the matching of cultivar and *Escovopsis *phylogenies at some scales. Discordance of host-parasite phylogenies, however, arises due to host-switching (Figure 3). These results reveal the need for additional sampling across the fungus-growing ant microbial symbiosis as a whole. To date, there has not been extensive sampling and analysis of the pathogens that attack the diverse G3 cultivars grown by many fungus-growing ant species [13]. There are also few published genetic analyses of the cultivars of the leafcutter ants, agricultural pests in much of the Neotropics, and the leafcutter-associated *Escovopsis*. Broad sampling and genetic analyses across the symbiosis will give insight into how labile these associations are over both ecological and evolutionary time.

## Methods

### Collections and infection prevalence

From 2001–2004, we sampled 632 *Apterostigma *colonies collected across their geographic range in order to isolate fungal symbionts (cultivar and *Escovopsis*). All fungi were cultured following procedures of [16]. *Escovopsis *samples from Panama, Costa Rica, and Argentina were maintained as live cultures on potato dextrose agar with 50 mg/L each of penicillin and streptomycin until spores and mycelium could be directly frozen at -80 degrees. Fungal samples from Ecuador were only temporally maintained live after collection and were then stored in 95% alcohol prior to export from the country. DNA extraction of frozen samples followed a CTAB extraction protocol modified from [31].

Infection prevalence in the three colony-types (G2, G3 and G4) was determined by dividing the number of colonies infected with *Escovopsis *by the total number of colonies from which either *Escovopsis *or cultivar was successfully isolated (colonies from which no microbes were isolated were excluded from these analyses). We then used log-likelihood ratio tests (a.k.a. G-tests) to compare rates of infection across colony-types. These tests were performed in R (ver 2.3.1, [32]) using the function g.test.r [33] with the William's correction applied.

### Samples for phylogenetic reconstruction

To determine the relationship amongst *Escovopsis *strains isolated from *Apterostigma *spp. colonies, samples for phylogenetic reconstruction were selected to include all *Escovopsis *morphotypes isolated from *Apterostigma *spp. colonies. Because colonies with G2 cultivars are commonly found and are frequently infected with *Escovopsis*, we sequenced more *Escovopsis *strains from G2 (n = 44) than from G3 (n = 5) or G4 (n = 4) colonies. We also sequenced one yellow-spored *Escovopsis *sample isolated from a *Cyphomyrmex longiscapus *colony for comparison with other yellow *Escovopsis *included this study. Sequencing targeted a 987 nucleotide stretch spanning 1 exon of nuclear elongation factor-1 alpha (EF-1 α) using PCR primers EF1-983F and EF1-2218 as well additional internal sequencing primers EF1-6mf and EF1-6mr [16]. All sequences are deposited in Genbank [GenBank:DQ848156 – DQ848209].

In the final alignment, we included five previously sequenced *Apterostigma*-associated *Escovopsis *[GenBank:AY172618, GenBank:AY172619, GenBank:AY629395–AY629397] as well as sequences of *Escovopsis *isolated from colonies of other fungus-growing ant genera [GenBank:AY172616, GenBank:AY172617, GenBank:AY172620, GenBank:AY172630, GenBank:AY172631, GenBank:AY629363, GenBank:AY629366, GenBank:AY629368, GenBank:AY62969, GenBank:AY629376, GenBank:AY629390]. For outgroups, we included sequences of *Aphysiostroma stercorarium *[GenBank:AF543782], *Bionectria ochroleuca *[GenBank:AY489611], *Cordyceps taii *[GenBank:AF543775], *Hypocrea lutea *[GenBank:AF543781], *Hypomyces polyporinus *[GenBank:AF543784], *Metarhizium anisopliae *[GenBank:AF543774], *Nectria cinnabarina *[GenBank:AF543785], *Ophionectria trichospora *[GenBank:AF543779], *Pseudonectria rousseliana *[GenBank:AF543780], *Rotiferophthora anguistispora *[GenBank:AF543776], *Sphaerostilbella berkeleyana *[GenBank:AF543783] and *Trichoderma *sp. [GenBank:AY629398]. For simplicity, not all of these outgroups are presented in the phylogram in Figure 2. Sequences were assembled in SeqMan II (ver 5.05, DNASTAR), aligned using Clustal W WWW [34]and edited manually in MacClade (ver 4.06, [35]).

### Phylogenetic analyses and hypothesis testing

Parsimony analyses were performed in PAUP* (ver 4.0b10, [36]) using heuristic searches with TBR branch swapping and 10,000 random addition sequence replicates (multrees = yes). In order to obtain estimates of clade support, non-parametric bootstrapping was performed with heuristic searches of 5000 replicate datasets and 10 random addition sequence replicates per dataset (multrees = no).

For maximum likelihood and Bayesian analyses, a model of sequence evolution was estimated for the data set using MODELTEST ver. 3.7 [37]. The chosen model, K81uf + pinvar + Γ, was used for all maximum likelihood analyses and parametric hypothesis testing. Because it is not possible to implement this model in Mr. Bayes, a more complex model of sequence evolution, GTR + pinvar + Γ, was used in all Bayesian analyses.

For maximum likelihood analysis, we performed a successive approximation search using PAUP* to estimate the topology [38]. Starting parameter values estimated from a parsimony tree (TBR branch swapping, 100 random addition sequence replicates, multrees = no) were used in an initial maximum-likelihood search. Parameters were then re-estimated from the resulting tree and the search was repeated with these new parameters. This procedure was repeated until the resulting tree was identical in topology to that from the previous iteration. Non-parametric bootstrapping was performed with heuristic searches of 1000 replicate datasets starting from a neighbor-joining tree (multrees = yes).

For Bayesian analyses, using Mr. Bayes (ver 3.0b4, [39]), four separate Markov Chain Monte Carlo (MCMC) runs were performed starting from random trees for each of four simultaneous chains. Runs were five million generations with a burn-in of 100,000 generations, default prior distribution for model parameters, and the differential heating parameter set to 0.2. The joint posterior probabilities and parameter estimates of each run were congruent, suggesting the chains were run for a sufficient number of generations to adequately sample the posterior probability landscape.

Phylogenetic analysis with no topological constraints indicated two origins of G4-associated *Escovopsis *(Figure 2). To test the hypothesis of monophyly of *Escovopsis *isolated from G4 colonies, we compared the observed, optimal tree (alternative hypothesis) to trees constrained to represent the null hypothesis of a single origin of G4 *Escovopsis*. Sequence evolution parameters were estimated by using maximum likelihood under the K81uf + pinvar + Γ. We used parametric bootstrapping procedures to evaluate 500 simulated datasets generated using Seq-Gen (ver 1.2.5, [40]).

## Authors' contributions

All authors organized fieldwork and collected colonies. NMG and CRC isolated, maintained and stored fungal samples. NMG performed molecular work and analyzed data. UGM and CRC contributed reagents/materials/analysis tools. NMG wrote the paper. All authors read and commented on drafts of the manuscript, and approved the final manuscript.
